# Case report: A novel SON mutation in a Colombian patient with ZTTK syndrome

**DOI:** 10.3389/fgene.2023.1183362

**Published:** 2023-07-05

**Authors:** Diana Marcela Vasquez-Forero, Barbara Masotto, Rosario Ferrer-Avargues, Christian Martin Moya, Harry Pachajoa

**Affiliations:** ^1^ Facultad de ciencia de la salud, Universidad Icesi, Cali, Colombia; ^2^ Departamento de Genética, Fundacion Valle del Lili, Cali, Colombia; ^3^ Medical Genetics Unit, Sistemas Genómicos, Paterna, Spain; ^4^ Centro de Investigaciones en Anomalías Congénitas y Enfermedades Raras Universidad Icesi, Cali, Colombia

**Keywords:** SON, Zhu–Tokita–Takenouchi–Kim syndrome, ZTTK syndrome, rare disease, Colombia

## Abstract

Zhu–Tokita–Takenouchi–Kim syndrome is a multisystem disorder resulting from haploinsufficiency in the SON gene, which is characterized by developmental delay/intellectual disability, seizures, facial dysmorphism, short stature, and congenital malformations, primarily in the central nervous system, along with ophthalmic, dental, pulmonary, cardiologic, renal, gastrointestinal, and musculoskeletal anomalies. In this study, we describe the first Colombian patient with ZTT harboring a novel mutation that has not been previously reported and review the clinical and molecular features of previously reported patients in the literature.

## Introduction

Zhu–Tokita–Takenouchi–Kim (ZTTK) syndrome is a rare autosomal dominant hereditary disease with multisystemic manifestations, caused by pathogenic variants in the SON gene, which is located on chromosome 21q22.11. The syndrome was first reported in 2015 by Zhu et al. (2015), who analyzed 119 trios based on Whole Exome Sequencing (WES) of patients with undiagnosed genetic diseases and identified a frameshift mutation in the SON gene in a 5-year-old girl with developmental delay, epilepsy, megalencephalic white matter dysplasia, mild dysmorphisms, intestinal atresia, and ventricular septal defect. Later in 2016, Takenouchi et al. (2016) reported a 13-year-old boy with the same frameshift mutation in SON, who had macrocephaly, facial dysmorphisms, severe intellectual disability, growth deficiency, and aortic valve regurgitation, but without brain abnormalities. That same year, Tokita et al. (2016) described seven individuals between 3 and 23 years of age with *de novo* truncating variants in SON, two of them with the previously reported frameshift mutation, presenting with developmental delay, short stature, hypotonia, and congenital malformations in the brain, heart, lungs, kidneys, and gastrointestinal tract.

In 2016, Kim et al. (2016) recruited 20 non-related individuals with *de novo* mutations in the SON gene and observed that all patients exhibited intellectual disability and facial dysmorphisms, with 17 of them also presenting with brain abnormalities, seizures or hypotonia, and other associated musculoskeletal, eye and vision, heart, urogenital, and gastrointestinal malformations. Thereby, ZTTK syndrome was named after the first authors who correlated the phenotypic findings with the pathogenic mutation in the SON gene (Tan et al., 2020).

Recently, several reports have contributed to establishing the phenotypic spectrum related to ZTTK syndrome. Dingemans in 2022 described 17 new patients who displayed the previously reported multisystem malformations, along with new abnormalities of the nails, skin, and hair, and also confirmed the presence of renal malformations and growth hormone deficiency in these patients (Dingemans et al., 2022). Additionally, in 2022, Kushary reported that hearing loss is an important feature observed in two out of 15 newly identified individuals with ZTTK syndrome, as well as in three previously reported patients (Kushary et al., 2021).

Here, we report the first case of ZTTK syndrome in Latin America, a 3-year-old boy with a *de novo* non-sense mutation in the SON gene, never reported in the literature.

## Case description

The proband is a 3-year-old boy, who is the only offspring of healthy, non-consanguineous parents of advanced age (mother aged 39 years and father aged 46 years). The pregnancy was uneventful, with no abnormalities detected in ultrasound scans. However, due to fetal distress, the child was delivered via Cesarean section at 29 weeks of gestation. At birth, the child weighed 700 g and measured 33 cm in length. He required hospitalization for 3 months due to bronchopulmonary dysplasia, neonatal feeding difficulties, and severe hypotonia. Cardiac imaging revealed no abnormalities. Additionally, he exhibited developmental delay and experienced seizures. During the first year of life, he had three episodes of pneumonia and gastroesophageal reflux. At the age of 2, a brain MRI was conducted due to persistent seizures, which revealed agenesis of the corpus callosum; an EEG reported an inappropriate configuration of the background rhythm, and as a result, was evaluated by a pediatric neurologist who diagnosed focal structural epilepsy with clones on the left side of the face during sleep. Treatment was initiated with levetiracetam, which successfully controlled the seizures.

**FIGURE 1:**
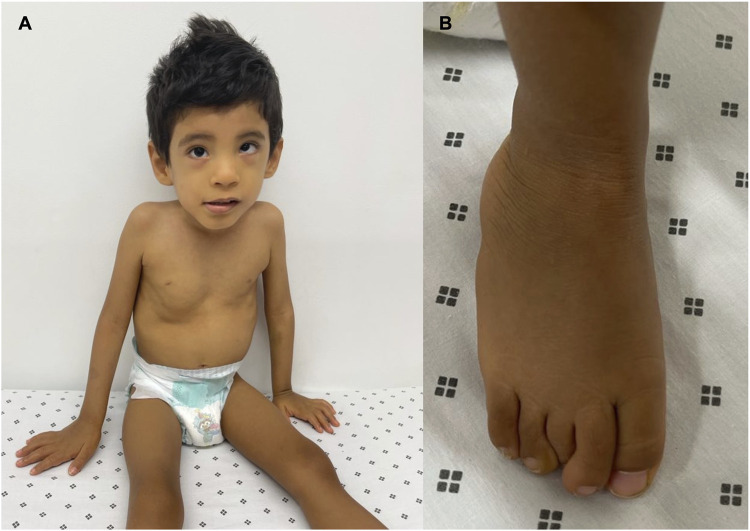
Phenotypic manifestations: **(A)** dysmorphic features such as hypertelorism, epicanthal folds, smooth philtrum, and depressed nasal bridge. **(B)** Superposition of the second and fifth finger over the third and fourth finger on the left foot.

The patient underwent a medical genetic consultation at the age of 3. The child weighed 8.5 kg (−5.85 SD), had a height of 90 cm (−3.09 SD), and had an OFC of 48.5 cm (−2.08 SD). Physical examination revealed several dysmorphic features, including plagiocephaly, hypertelorism, hyperesotropia of the left eye, epicanthal folds, smooth philtrum, depressed nasal bridge, bilateral clinodactyly of the fifth finger, and superposition of the second and fifth finger over the third and fourth finger on the left foot. Moreover, he presented with inguinal hernia, non-descended testicles, and phimosis (Figure 1). Additionally, the patient presented with severe psychomotor development delay, stereotyped movements, persistent bruxism, generalized hypotonia, no interaction with the interlocutors, lack of gaze fixation, and absence of language.

The proband’s karyotype was normal for a male. The chromosome microarray analysis did not reveal any chromosomal numerical abnormalities or pathogenic CNVs. The underlying cause was unknown, so a trio whole-exome analysis was performed.

**FIGURE 2:**
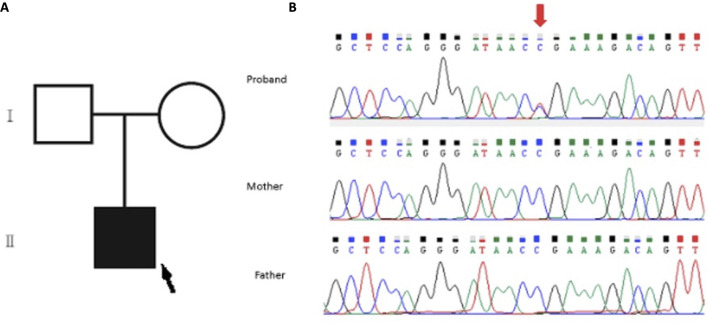
**(A)** Family pedigree of the patient: the proband is indicated with a black arrow. **(B)** Sanger sequencing analysis of the family. Upper panel (proband): segments of genomic DNA sequences showing the diagnostic variant SON:c.5743C>T indicated by the red arrow. Note the absence of such a variant in the middle and lower panel for the father and mother, respectively.

The WES analysis revealed an average read depth of 133.25x for this sample, and the coding regions of the genes included in this test had a coverage (≥20x) of 97.44%. A pathogenic, *de novo*, heterozygous variant SON: c.5743C>T; p.Arg1915Ter was identified in the patient and confirmed by Sanger sequencing (Figure 2). The parents tested negative.

## Material and methods

### Chromosome karyotyping

The karyotype was determined in the child. A blood sample of 0.3 mL was incubated in a culture medium for complete separation of lymphocytes (20% fetal calf serum in Roswell Park Memorial Institute 1640 medium with 2.5% phytohemagglutinin and 2% L-glutamine and 1% penicillin–streptomycin in an incubator at 37°C for 72 h). Metaphases were harvested by adding colcemid for 60 min, followed by the addition of hypotonic potassium chloride (0.075 M) (treatment for 10 min and fixation, using standard 3:1 methanol–acetic fixative—Merck). Chromosome preparations were stained with G-banding. Chromosomal analysis revealed 46, XY.

### DNA extraction

Peripheral blood samples were obtained from the proband and his parents. The genomic DNA was extracted using a Qiagen QIAamp DNA Mini Kit according to the manufacturer’s protocol.

### Chromosomal microarray analysis

The Affymetrix CytoScan 750 K gene chip (Applied Biosystems) was used for DNA digestion, amplification, purification, fragmentation, labeling, hybridization, rinsing, staining, and scanning. ChAS software was used to analyze the scanned data. The established size threshold for reporting copy number variants (CNVs) was ≥100 Kb for gains and losses and ≥5 Mb for reduction of heterozygosity (ROH).

### Whole exome sequencing (WES)

Genomic DNA of the proband and his parents was sequenced using next-generation sequencing (NGS). A trio whole exome sequencing was performed. Target regions were captured using the SureSelectXT Human All Exon V6 (Agilent Technologies), and sequencing was performed on an Illumina NovaSeq, with a read length of 2 × 150 bp.

The sequencing reads were aligned to the human reference genome (GRCh38) using the Burrows–Wheeler Aligner. The Genome Analysis Toolkit (GATK) was used to perform variant calling (VCF_files). Variants were filtered using GeneSystems^®^ analysis software according to their predicted effects, allele frequencies, with a read count≥ 10x, and a variant reads/total reads ratio ≥ 0.2. The analyzed regions include the coding exons and adjacent intronic regions (±8bp) of the captured genes. The Integrative Genomics Viewer was used for visual exploration of the clinically relevant variants.

The American College of Medical Genetics and Genomics (ACMG) criteria was used for variant classification (Richards et al., 2015).

### Sanger sequencing

Clinically relevant variants were confirmed by Sanger sequencing in the proband and his parents. The 3730xl DNA Analyzer (Applied Biosystems) was used, and the sequence of the primers was as follows: forward 5′ TTC​ACG​GTC​ACG​TTC​AAG​AC and reverse 5′AAA​GAC​AGA​AAC​ATT​TAT​CCT​AAA​CCA.

## Discussion

Zhu–Tokita–Takenouchi–Kim syndrome is an autosomal dominant hereditary disease characterized by facial dysmorphisms, short stature, intellectual disability/development delay, and hypotonia, often accompanied by congenital malformations affecting the brain, heart, kidneys, gastrointestinal tract, and musculoskeletal system. To date, 70 patients, including our patient, have been reported with a pathogenic frameshift mutation in SON, and they exhibit similar phenotypes, including dysmorphic features, developmental delay/intellectual disability (91.5%), hypotonia (65.7%), and brain malformation (77%) such as ventricular enlargement, leukodystrophy, cerebellar dysplasia, and corpus callosum abnormality, with or without the presence of seizures and hypotonia, EEG abnormalities, regression, and an autism spectrum disorder (Tokita et al., 2016). Eye abnormalities (52.8%) described are strabismus, myopia, and hypermetropia (Dingemans et al., 2022) (Slezak et al., 2020), while hearing loss (11.5%) is the main otorhinolaryngological feature, typically moderate to severe, and sometimes congenital (Kushary et al., 2021). Heart malformations (21.4%) include septal defects, aberrant subclavian artery, valvopathies, and patent ductus arteriosus (Tokita et al., 2016). Concomitant gastrointestinal malformations (18.5%) include intestinal atresia and duodenal malformation (Dingemans et al., 2022), while urogenital malformations (24.2%) such as single kidney, horseshoe kidney, and kidney dysplasia have been reported (Dingemans et al., 2022) (Slezak et al., 2020). Musculoskeletal abnormalities (68.5%) include short stature, craniosynostosis, joint laxity, cubitus valgus, scoliosis, hemivertebrae, cervical cord compression, lumbar hyperlordosis, contractures, small hands and feet, flat feet, aplasia/hypoplasia of the thumb, genu valgum, and abnormal rib defects (Slezak et al., 2020). Skin, hair, and nail alterations (15.7%) and teeth alterations (4.3%) such as retronychia, onychodystrophy, hyperkeratosis, dental enamel hypoplasia, and microdontia have also been reported (Table 1) (Quintana Castanedo et al., 2020).

**TABLE 1:** Phenotype summary of clinical features of ZTTK syndrome.

Characteristic	Present report	Number	%
Female	-	33	47
Male	+	37	52.8
Short stature	+	16	23
Microcephaly	+	11	16
Neonatal feeding difficulties	+	37	52.8
Intellectual disability	+	64	91.5
**Brain malformation**	+	54	77
Ventricular enlargement	-	36	51.4
Corpus callosum abnormality	+	28	40
Cortex malformation	+	14	20
White matter abnormalities	-	7	10
Cerebellum abnormalities	-	5	7
Seizures	+	37	52.8
Hypotonia	+	46	65.7
**Musculoskeletal abnormalities**	+	48	68.5
Hypermobility	+	25	35.7
Scoliosis/kyphosis—Vertebral	+	9	13
Hemivertebrae	-	4	6
Contractures	-	6	8.5
**Eye/vision abnormality**	+	37	52,.8
Strabismus	+	24	34.2
Suspicion of cerebral visual impairment	-	4	6
Hypermetropia	+	14	20
**Heart defect**	-	15	21.4
Ventricular septal defect	-	5	7
Atrial septal defect	-	8	11.4
Valve alteration	-	3	4.3
Artery malformation	-	3	4.3
**Gastrointestinal malformation**	-	13	18.5
Intestinal atresia	-	3	4.3
Gallbladder agenesis	-	1	1.4
Intestinal malrotation	-	3	4.3
**Urogenital malformation**	-	17	24.2
Single kidney	-	1	1.4
Dysplastic kidney	-	7	10
Horseshoe kidney	-	5	7
Polycystic kidneys	-	2	3
Pyelectasis	-	3	4.3
Undescended testis	+	2	3
Recurrent UTIs	-	1	1.4
**Pulmonary malformation**	+	2	3
Bronchodysplasia	+	0	0
Agenesis of the left lung	-	1	1.4
**Skin**–**hair**–**nail abnormality**	-	11	15.7
Teeth abnormality	-	3	4.3
**Hearing impairment**	-	8	11.5
Facial dysmorphism	+	46	66
Craniosynostosis	+	6	8.5
Growth hormone deficiency	-	4	5.7
Ig deficiency	-	6	8.5

Furthermore, certain authors have reported on psychiatric conditions that were not previously included in the spectrum of the disease, but are present in most patients, such as autism spectrum disorder, destructive behavior, severe maladjusted behavior, hyperactivity, and neural regression (Yang and Yang, 2020). A small number of cases have also been noted to exhibit feeding difficulties after birth (52.8%): dysphagia, poor suckling, gastroesophageal reflux, and immunoglobulin deficiency (8.5%); mainly IgA deficiency (Tokita et al., 2016); as well as growth hormone deficiency (5.7%) (Slezak et al., 2020).

The principal clinical features of our patient overlap with those previously reported. However, it is noteworthy that our patient presented with bronchodysplasia, and pulmonary manifestations have only been reported in one other patient with agenesis of one lung (Tokita et al., 2016). While a specific facial dysmorphism for ZTTK syndrome has not been established yet, we observed some recurrent characteristics such as microcephaly and craniosynostosis, which have been reported in 16% and 8.5% of previously reported patients, respectively; other features that were observed include hypertelorism, epicanthal folds, strabismus, smooth philtrum, and thin lips.

The SON gene is located on the chromosome 21q22.11 and consists of 34,478 base pairs (bp) distributed across 12 exons. Exon 3 constitutes 82% of the gene’s coding region (Yang et al., 2019). The gene comprises three significant domains: an arginine/serine-rich domain, a G-patch domain, and a double-stranded RNA-binding motif (Tokita et al., 2016). SON is a nuclear speckle-localized protein that functions as a splicing co-factor and participates in the regulation of cell cycle progression. It plays a crucial role in neural development (Yang and Yang, 2020). Studies conducted in murine animal models have shown that SON knockdown causes alterations in neuronal migration and decreases the dendritic spine density in the cerebral cortex (Ueda et al., 2020). Additionally, in zebrafish models, haploid deficiency of the SON gene causes alteration in the tail length and body axis, associated with eye malformation and microcephaly (Kim et al., 2016). The SON gene is also recognized as a key neurodevelopment regulator, controlling the expression of other neurodevelopment, metabolism, and neuronal cell transition genes (Yang et al., 2019).

Until now, the 70 cases described in the literature reported 53 different mutations in SON. Of these mutations, 33 are frameshift mutations, with the most common mutations being c.5753_5756del and p (Val1918GlufsTer87), located in the exon 3 on the arginine/serine rich domain, which may have a genotype–phenotype correlation with cardiac defects (Kushary et al., 2021); Additionally, there were four missense, two in-frame deletions, eight non-sense variants, a 0.19 Mb deletion, and a whole-gene deletion.

The characteristic phenotype was observed in the individuals with a variant that causes loss of function in SON, leading to haploinsufficiency (Dingemans et al., 2022) (Table 2). Although patients with a missense variant present with a similar phenotype, at the cellular level, it does not cause neuronal migration defects, indicating that the pathophysiological mechanisms are different and are yet to be described (Dingemans et al., 2022).

**TABLE 2:** Genotype summary of mutation types identified in SON (NM_138927.4) in association with ZTTK syndrome reported in the literature to date.

cDNA variant	Protein change	Frequency
c.5753_5756del	p.Val1918GlufsTer87	17
c.3852_ 3856del	p.Met1284IlefsTer2	2
c.1881_1882del	p.Val629AlafsTer56	2
c.6010del	p.Val2004TrpfsTer2	2
c.3334C>T	p.Arg1112Ter	2
c.5297del	p.Ser1766LeufsTer7	1
c.5230delC	p.Arg1744ValfsTer29	1
c.6233delC	p.Pro2078HisfsTer4	1
c.3073dupA	p.Met1025AsnfsTer6	1
c.1444del	p.Leu482CysfsTer4	1
c.4018delG	p.Ala1340GlnfsTer26	1
c.4919_4923del	p.Asp1640GlyfsTer7	1
c.5549_5550del	p.Arg1850IlefsTer3	1
c.5031_5032insAA	p.Asp1678LysfsTer9	1
c.6002_6003insCC	p.Arg2002GlnfsTer5	1
c.4358_4359del	p.Thr1453SerfsTer11	1
c.4640del	p.His1547LeufsTer76	1
c.6087del	p.Ser2029ArgfsTer22	1
c.3597_3598dup	p.Pro1200ArgfsTer17	1
c.2365del	p.Ser789AlafsTer8	1
c.268del	p.Ser90ValfsTer59	1
c.4055del	p.Pro1352GlnfsTer14	1
c.4549dup	p.Glu1517GlyfsTer6	1
c.457del	p.Asp153IlefsTer4	1
c.384del	p.Lys128AsnfsTer21	1
c.3711del	p.Ser1238GlnfsTer3	1
c.348_351del	p.Asn116LysfsTer32	1
c.326_329del	p.Lys109SerfsTer39	1
c.3135_3157del	p.Glu1046GlyfsTer2	1
c.6461del	p.Asn2154IlefsTer2	1
c.4776_4779del	p.Ser1594LeufsTer28)	1
c.4777_4778del	p.Leu1593IlefsTer11	1
c.4448_4452del	p.Val1483GlufsTer4	1
c.2160delC	p.Met721TrpfsTer6	1
c.4663delA	p.Thr1555LeufsTer68	1
c.4999_5013del	p.Asp1667_Asn1671del	1
c.4151_4174del	p.Leu1384_Val1391del	1
c.394C>T	p.Gln132Ter	1
c.286C>T	p.Gln96Ter	1
**c.5743C>T**	**p.Arg1915Ter**	1
c.3203C>G	p.Ser1068Ter	1
c.3408C>A	p.Tyr1136Ter	1
c.5761C>T	p.Arg1921Ter	1
c.2763C​>A	p.Tyr921Ter	1
c.4909A>T	p.Thr1637Ser	1
c.5528C>A	p.Ser1843Tyr	1
c.668C>T	p.Ser223Leu	1
c.3214C>T	p.Arg1072Cys	1
Whole-gene deletion	—	1
0.19 Mb deletion	—	1
—	TOTAL	70

^a^ All variants were found “*de novo.*”

The bold values are the ones present in our patient.

Our patient presents with a non-sense variant: c.5743C>T (p.Arg1915Ter), causing a truncated protein; according to the ACMG guidelines (Richards et al., 2015), the mutation is classified as a pathogenic mutation, which had not been previously reported in the literature, the first described case being the ZTTK case in Colombia.

After the diagnosis, paraclinical tests were conducted to investigate the phenotypic findings described in previous reports. However, these tests did not reveal any additional abnormalities in the gastrointestinal tract, renal system, or hearing ability. Since the seizures were well-managed with levetiracetam, no adjustments were made to the medication.

Concluding is important to recognize ZTTK syndrome as a severe multisystem developmental disorder suspected in patients with developmental delay/intellectual disability, short stature, facial dysmorphism, and congenital malformations: neuronal, ophthalmological, otorhinolaryngological, dental, pulmonary, cardiological, renal, gastrointestinal, and musculoskeletal. The diagnosis is established in a proband with suggestive findings and loss-of-function variants in the SON gene.

## Data Availability

The datasets for this article are not publicly available due to concerns regarding participant/patient anonymity. Requests to access the datasets should be directed to the corresponding author.
